# An Automated Method To Predict Mouse Gene and Protein Sequences Using Variant Data

**DOI:** 10.1534/g3.119.400983

**Published:** 2020-01-07

**Authors:** Peter Dornbos, Anooj A. Arkatkar, John J. LaPres

**Affiliations:** * Department of Biochemistry and Molecular Biology and; † Institute for Integrative Toxicology, Michigan State University, East Lansing, Michigan

**Keywords:** Amino Acid Imputation, Gene Imputation, *Mus Musculus*, Mouse Genetics, Aryl Hydrocarbon Receptor

## Abstract

With recent advances in sequencing technologies, the scientific community has begun to probe the potential genetic bases behind complex phenotypes in humans and model organisms. In many cases, the genomes of genetically distinct strains of model organisms, such as the mouse (*Mus musculus)*, have not been fully sequenced. Here, we report on a tool designed to use single-nucleotide polymorphism (SNP) and insertion-deletion (indel) data to predict gene, mRNA, and protein sequences for up to 36 genetically distinct mouse strains. By automated querying of freely accessible databases through a graphical interface, the software requires no data and little computational experience. As a proof of concept, we predicted the gene and amino acid sequence of the aryl hydrocarbon receptor (*Ahr*) for all inbred mouse strains of which variant data were currently available through Mouse Genome Project. Predicted sequences were compared with fully sequenced genomes to show that the tool is effective in predicting gene and protein sequences.

In the last several decades, the genomes of many different organisms have been sequenced, assembled, and annotated. While DNA-sequencing technology continues to advance rapidly, whole-genome sequencing and data processing for assembly and annotation are not always feasible. Genetic differences across strains of *Mus musculus* are known to drive a plethora of strain-specific phenotypic responses. For example, alterations in the *Kras* gene within the A/J strain likely play a role in lung tumor susceptibility (Chen *et al.* 1994; You *et al.* 1993). Similarly, NOD mice are genetically prone to developing type-1 diabetes (Thayer *et al.* 2010). While some mouse genomes have been fully-sequenced (Doran *et al.* 2016; Keane *et al.* 2011; Wong *et al.* 2012; Yalcin *et al.* 2011), many remain poorly characterized. As such, genetic sequence imputation has been used extensively in the past (Ellinghaus *et al.* 2009; Nothnagel *et al.* 2009).

During a previous toxicology study, we sought to establish and compare the impacts of genetic variation within the amino acid sequence of several proteins across a large number of strains which have not been fully-sequenced (Dornbos *et al.* 2018). While doing so, we found a lack of software to predict strain-specific impacts of variations in gene and protein sequences in a high-throughput manner. While many software packages predict the impact of variation including the widely-used Ensembl Variant Effect Predictor (VEP)(McLaren *et al.* 2016; Pabinger *et al.* 2014), packages that support the mouse genome require users to provide variant call data and lack support to run multiple strains in parallel. Hence, we developed the mouse gene and protein sequence predictor (MGP-Seq). MPG-Seq is written in Python3 and designed to impute polymorphism data, include single nucleotide polymorphisms (SNPs) and indels, based on the reference mouse genome. The software utilizes the NCBI Gene database and Sanger’s Mouse Genome Project variant querying tool to predict gene, mRNA, and/or amino acid sequences in automated fashion requiring no variant call data from the user. MGP-Seq can impute data for any strain of which variant data are available on the Mouse Genome Project website. Finally, a graphical user interface (GUI) was implemented to ensure the user does not need experience working at a command-prompt (Figure 1). We believe this tool will be useful to researchers interested in examining the impact of genetic variation in mice at the gene and amino acid sequence level with potential to enlighten variant-to-function relationships.

**Figure 1:**
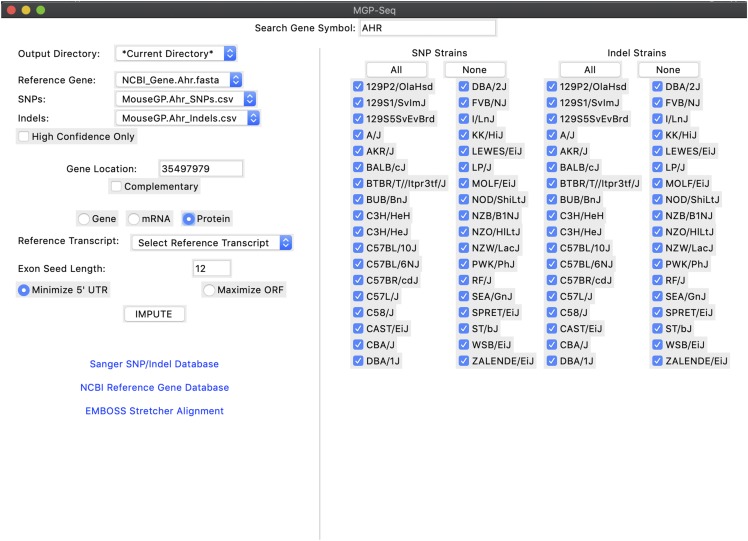
Screenshot of the graphical user interface for MGP-seq. The screenshot indicates the user’s view following the automated download of a) reference gene sequence and b) variant call data (SNPs and Indels). The interface allows user to select ‘all’ or strains and impute the gene, mRNA, or protein sequence.

As a proof-of-principle, we report an analysis of sequence variation in the murine aryl hydrocarbon receptor (*Ahr*), which encodes a ligand-activated sensory protein within the PAS superfamily (Abel and Haarmann-Stemmann 2010; Sorg 2014; Swanson *et al.* 1995). AHR-activation is linked to several complex diseases including immunotoxicity, metabolic syndrome, and diabetes (Uemura *et al.* 2009; Warner *et al.* 2013; Dornbos *et al.* 2016). Previous reports have indicated that mice carry four structurally and functionally unique alleles of the *Ahr*: 1) *Ahr*^b1^, 2) *Ahr*^b2^, 3) *Ahr*^b3^, and 4) the *Ahr*^d^ (Poland *et al.* 1994; Thomas *et al.* 2002; Poland and Glover 1990). Here, we use MGP-seq to predict genomic and amino acid sequences of the AHR for the 36 available strains through the Mouse Genomes Project. For a subset of strains, results from MGP-Seq were compared to fully sequenced genomic data to show the software’s reproducibility. Overall, MGP-Seq is an effective, and user-friendly tool for imputing mouse sequence variation into the reference genome.

## Methods

### Querying databases

**Figure 2:**
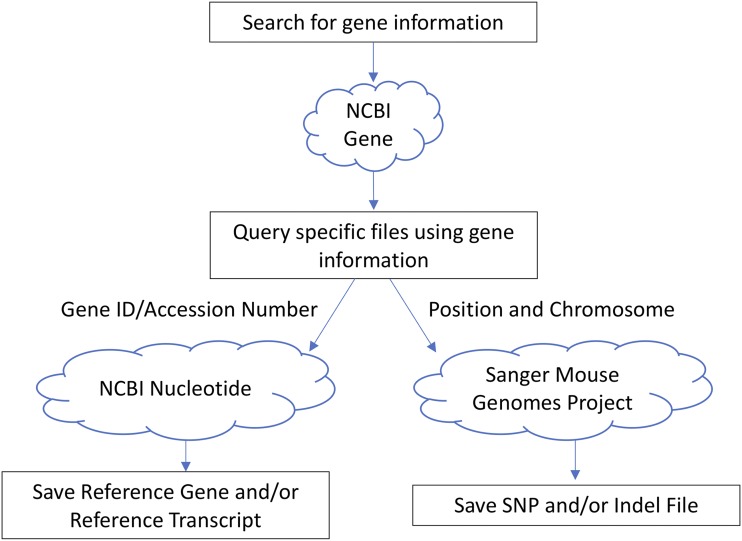
A flow-through diagram of the databases queries. Upon execution of the program, a GUI will prompt the user to enter an official gene symbol. The script then queries the NCBI Gene database for information on the gene entered, such as chromosome and position coordinates and whether it is encoded on the forward or compliment strand. The information gathered is used to download a) the reference gene sequence from the NCBI nucleotide database and b) variant call data (both SNPs and indels) from any mouse strain selected on the Sanger Mouse Genome website.

Reference gene and transcript sequences are queried using the NCBI Entrez API (Figure 2). Sequences are retrieved by the ESearch-EFetch pipeline (Sayers 2009). SNP and Indel data are requested through the Mouse SNP/Indel Viewer API from Sanger’s Mouse Genomes Project (Keane *et al.* 2011).

### Gene, mRNA, and protein prediction overview

#### Imputation:

**Figure 3:**
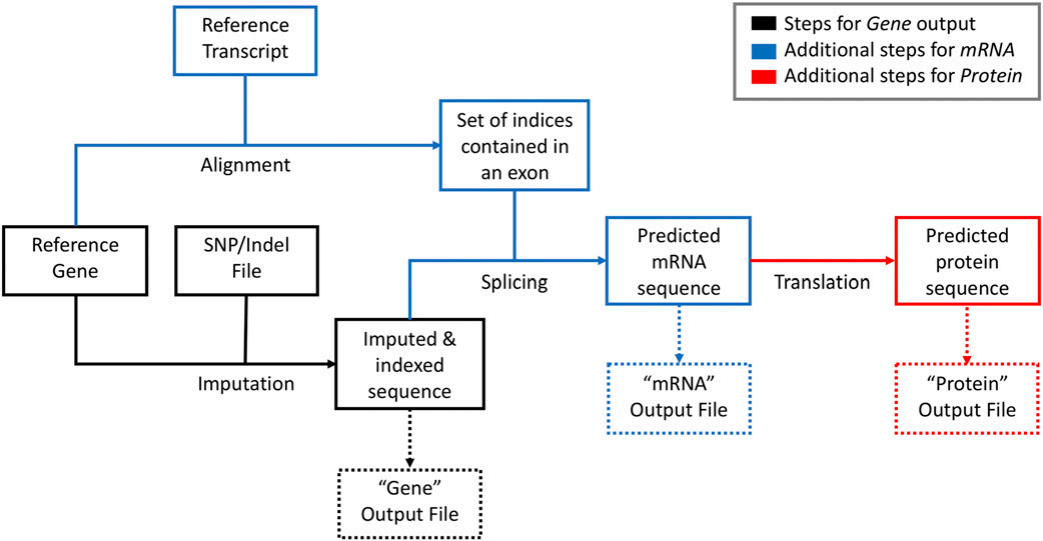
A flow-through diagram of the imputation process. Colors indicate steps necessary for the specified output type. Dashed lines represent possible output files. Upon clicking “Impute,” the SNP/Indel file will be used to impute strain-specific variations into the reference gene, using a method that retains the original index. An alignment algorithm identifies exonic indices to predict an mRNA sequence. It is then translated into the predicted protein sequence.

Figure 3 illustrates the process of sequence prediction. First, a hash table maps the index of the nucleotide to the listed SNPs and Indels. At a SNP, the nucleotide at the specified index is substituted for the variant nucleotide. At an insertion, this will result in an index containing more than one nucleotide. At a deletion, the deleted nucleotides are replaced by empty strings. This approach conserves the original index of the reference sequence.

#### Alignment to reference transcript:

The alignment is generated using the same general process outlined in the sim4 alignment algorithm (Florea *et al.* 1998). First, potential exons are seeded. Then, a dynamic programming algorithm selects the optimal set of exons. Finally, exon boundaries are identified. Exons are seeded using BLAT’s *Single Perfect Matches* search (Kent 2002). Dijkstra’s Shortest Path algorithm then selects the most probable set of exons with a cost function that attempts to minimize the number of mismatched mRNA nucleotides and the total number of introns (Dijkstra 1959). Finally, exon boundaries are selected such that preference is given to boundaries that match the GT/AG intron consensus. Remaining, unaligned mRNA bases are aligned using Gotoh’s Algorithm (Gotoh 1982).

#### Translation:

Translation proceeds using the standard codon table. There are two options for identifying the Open Reading Frame (ORF). The first option, “Minimize 5’-UTR,” returns the ORF that starts at the very first start codon. The second option, “Maximize ORF,” examines the ORF beginning with each potential start codon and translates the longest one.

### Genomic data acquisition and protein sequencing drediction during testing

To test the efficacy of MGP-Seq imputation, genomic data were downloaded from the Collaborative Cross website (https://csbio.unc.edu/CCstatus/index.py?run=Pseudo). Full genomes were downloaded for a) C57BL/6J, b) A/J, c) DBA/2J, and d) PWK/PhJ. The coordinates from the *Ahr* gene in the reference mouse genome were used to flank the *Ahr* gene in each strain. The *Ahr* gene was then aligned to the reference mRNA reported on NCBI-Gene to remove intronic regions. The exonic regions were used to predict the amino acid sequence for each strain using EMBOSS transeq (https://www.ebi.ac.uk/Tools/st/emboss_transeq/).

### Sequence alignments during testing

Pairwise sequence alignments for nucleotide and amino acid sequences were completed with the EMBOSS stretcher online tool (https://www.ebi.ac.uk/Tools/psa/emboss_stretcher/). Multiple sequence alignments (MSAs) were executed with the Multiple Alignment using Fast Fourier Transform (MAFFT) software (Katoh *et al.* 2005). In all cases, multiple sequence alignments were completed with global alignments with a maximum of 1000 iterations. Sequence alignments were compared visually using phylogenetic trees and with clustal formatted outputs.

### Phylogenetic tree analysis

Phylogenetic trees were built using the outputs from MAFFT global sequence alignments. The trees were visualized using FigTree software version 1.4.2 (http://tree.bio.ed.ac.uk/software/figtree/). In all cases, phylogenetic trees reported include a scale bar.

### Data availability

MGP-Seq is released under the MIT License. The software and its documentation are freely available at https://github.com/aarkatkar/MGP-Seq. Software requirements and a detailed explanation of running the script are found in the README.md file in the repository. The software itself is contained entirely in MGP-Seq.py.

## Results

### Comparison of Ahr sequences from imputed and full-sequenced genomes

Pairwise sequence-alignments were used to compare imputation results from MGP-Seq and reported genomic data from four inbred mouse strains that carry differing alleles of the *Ahr* gene: C57BL/6J (*Ahr*^b1^), A/J (*Ahr*^b2^), PWK/EiJ (*Ahr*^b3^), and DBA/2J (*Ahr*^d^). Across all strains, MGP-seq imputation provides a sequence that shares greater similarity to previously reported sequences than does the reference AHR gene (Table 1). For example, imputation in the A/J strain (*Ahr*^b2^) provides a sequence that is 1.3% (*i.e.*, ∼400 bases) more similar to the previously reported gene sequence.

**Table 1:** Comparison of imputed sequence to reference mouse and previously-reported data for the 4 differing *Ahr* alleles. Pairwise sequence alignments were used to compare the imputed genetic and amino acid sequence with previously-documented sequence data for each *Ahr* allele. Previously reported sequences were compared to 1) the sequence found with the reference mouse genome used as imputation template and 2) sequence predicted by the imputation software. We report the number of matched nucleotides or amino acid out of the total for each respective comparison followed by percent sequence similarity

		Genetic Sequence		Amino Acid Sequence	
Strain	*Ahr* Allele	Compared to Reference Sequence	Compared to Imputed Sequence	Compared to Reference Sequence	Compared to Imputed Sequence
**C57BL/6JN**	*Ahr*^b1^	37011/37011 (100.0%)	37011/37011 (100.0%)	805/805 (100.0%)	805/805 (100.0%)
**A/J**	*Ahr*^b2^	36534/37108 (98.5%)	36934/37023 (99.8%)	799/848 (94.2%)	848/848 (100.0%)
**PWK/EiJ^1^**	*Ahr*^b3^	36541/37087 (98.5%)	36898/37033 (99.6%)	800/848 (94.3%)	848/848 (100.0%)
**DBA/2J**	*Ahr*^d^	36530/37125 (98.4%)	36954/37007 (99.9%)	798/848 (94.1%)	805/805 (100.0%)

**Figure 4:**
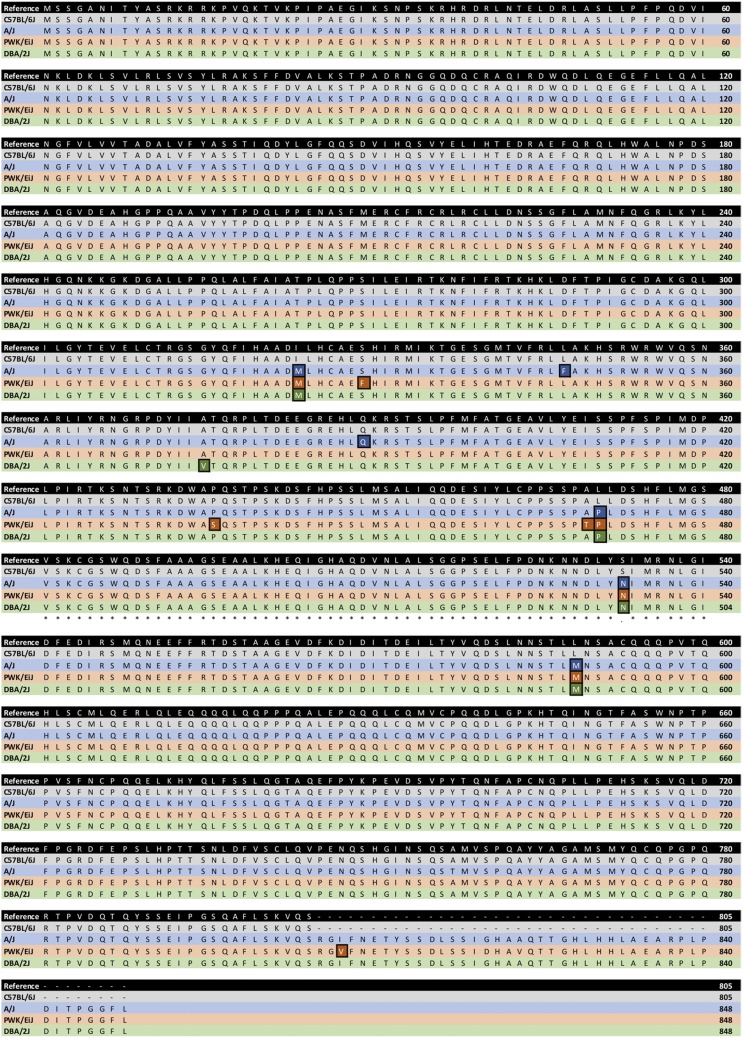
Multiple sequence alignment comparing the AHR amino acid sequence. Sequences inferred from the reference mouse genome (GRCm38) were compared with imputation results from 4 strains which carry differing alleles of the *Ahr*: C57Bl/6J (*Ahr^b1^*; gray), A/J (*Ahr^b2^*; blue), PWK/PhJ (*Ahr^b3^*; orange), and DBA/2J (*Ahr^d^*; green). Highlighted boxes indicate sequence variation. Black indicates the reference mouse sequence.

MGP-Seq proved similarly effective in predicting amino acid sequences. *Ahr* sequences derived from previous sequencing experiments of C57BL/6J (*Ahr*^b1^), A/J (*Ahr*^b2^), PWK/EiJ (*Ahr*^b3^), and DBA/2J (*Ahr*^d^) were obtained. After identifying and translating the exonic regions, the resultant amino acid sequences were then compared to those outputted by MGP-Seq (Table 1). Across all strains, the amino acid sequences predicted by MGP-Seq were identical to those predicted by gene sequencing. Finally, a multiple sequence alignment of the imputed amino acid sequence was used to show that MGP-Seq was capable of correctly identifying the well-established differences in the amino acid sequence (including single substitutions, insertions, and an early stop codon in the *Ahr*^b1^ allele) across the differing *Ahr* alleles (Figure 4).

### Comparison of Ahr genetic and protein sequence for 36 inbred mouse strains

To show that this software is scalable beyond analyzing single mouse strains, we imputed *Ahr* gene and amino sequences for each mouse strain (n = 37 if including the reference genome) of which there is polymorphism data on the Mouse Genome Project variant querying tool (Figure 1). Of particular note is the efficiency of the script; on average, imputation and prediction of amino acid sequence of the AHR for a single strain takes less than 0.04 sec while increasing to all 36 strains takes 0.22 sec on a personal computer with a 2.5 GHz Intel Core i5 processor and 8 GB of memory.

**Figure 5:**
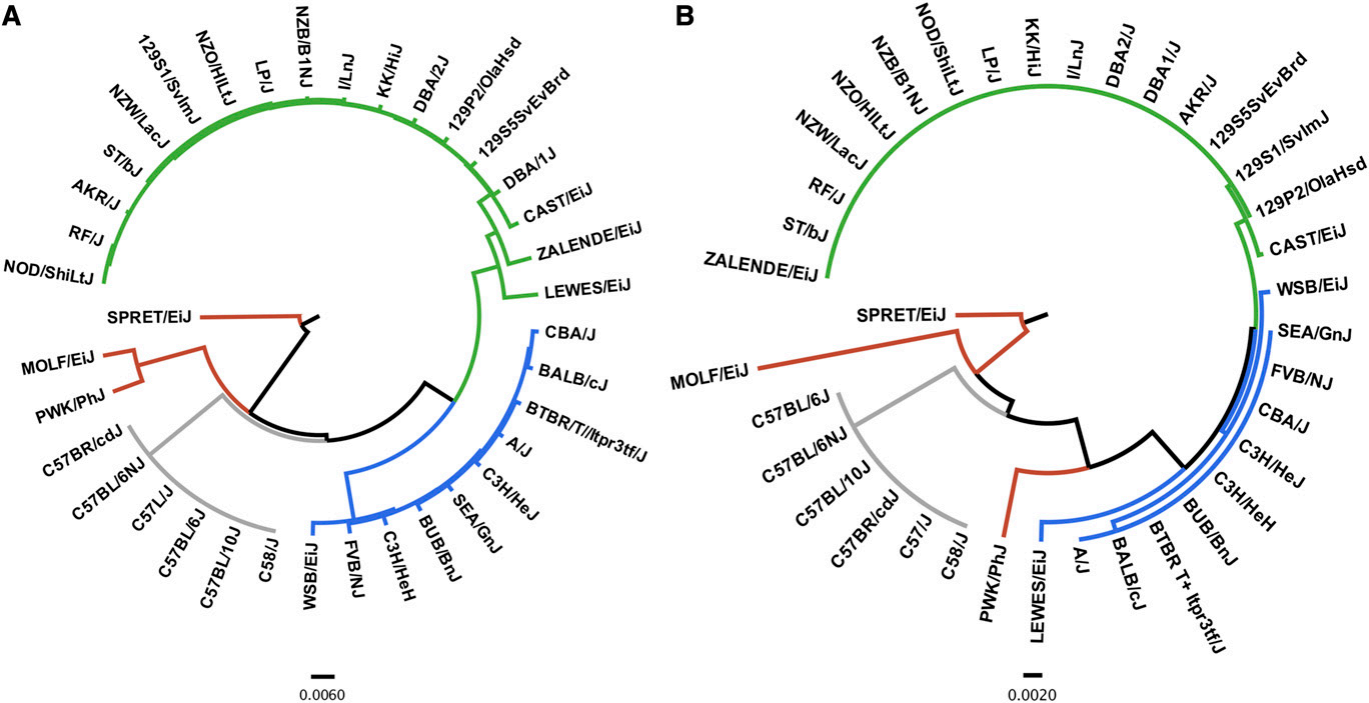
Phylogenetic analysis of the genetic and amino acid sequences for the *Ahr* of 37 inbred mouse strains. Trees were derived from a multiple sequence alignment of A) genetic sequences or B) amino acid sequences using the Multiple Alignment using Fast Fourier Transform (MAFFT) software (Katoh *et al.* 2005). Trees are rooted on SPRET/EiJ. Gray indicates *Ahr*^b1^ mice, blue indicates *Ahr*^b2^ mice, orange indicates *Ahr*^b3^ mice, and green indicates *Ahr*^d^ mice. Scale bar indicates relative distance for each tree.

The gene and amino acid sequence prediction output was used to highlight a potential use of the software. First, the *Ahr* sequence has not been previously characterized for 11 of the 36 inbred strains included in this study (Table 2). As such, this is the first report of which the *Ahr* sequence is predicted for a number of mouse strains. Second, a multiple sequence alignment of the genetic sequence across all strains indicates some interesting patterns (Figure 5A). Mouse strains with previously characterized *Ahr* alleles grouped most closely with other mice known to carry the same allele. In comparing allele frequencies across the 37 inbred strains, the majority of mice were found to carry the *Ahr^d^* allele (47.2%) while the least carry the *Ahr^b3^* allele (8.3%). Wild-derived strains (*i.e.*, SPRET/EiJ, MOLF/EiJ, PWK/PhJ, WSB/EiJ, and LEWES/EiJ) were found to carry the most polymorphic *Ahr* sequences. In contrast, there were no polymorphisms present with the *Ahr* sequence across all mice which carry the *Ahr^b1^* allele. Interestingly, the LEWES/EiJ strain was found to share more sequence similarities to mice which carry the *Ahr^d^* as compared to *Ahr^b2^* mice. However, based on genetic sequence, the LEWES/EiJ carries an *Ahr^b2^* allele as it lacks a SNP (rs3021544) which drives an A375V substitution that is unique to *Ahr^d^* allele mice.

**Table 2:** Predicted *Ahr* allele carried by 37 inbred mouse strains as inferred by imputation results. The *Ahr* allele carried by each strain was inferred based on similarity with known *Ahr^b1^*, *Ahr^b2^*, *Ahr^b3^*, *Ahr^d^* amino acid sequences. Letters indicate whether alleles have been previously reported

	*Ahr*^b1^	*Ahr*^b2^	*Ahr*^b3^	*Ahr*^d^
**Mouse Strains**	C57BL/6J*^a^*	A/J*^a^*	SPRET/EiJ*^a^*	129S1/SvImJ*^b^*
	C57BL/6NJ*^b^*	BALB/cJ*^b^*	MOLF/EiJ*^a^*	129S5SvEvBrD*^b^*
	C57L/J*^b^*	BTBR T+ Itpr3tf/J*^c^*	PWK/PhJ*^c^*	129P2/OlaHsd*^b^*
	C57BL/10J*^b^*	BUB/BnJ*^b^*		AKR/J*^b^*
	C57BR/cdJ*^b^*	C3H/HeJ*^a^*		DBA1/J*^c^*
	C58/J*^b^*	C3H/HeH*^b^*		DBA2/J*^a^*
		CBA/J*^b^*		I/LnJ*^b^*
		FVB/NJ*^c^*		KK/HiJ*^c^*
		SEA/GnJ*^b^*		LP/J*^b^*
		WSB/EiJ*^c^*		NOD/ShilLtJ*^c^*
		LEWES/EiJ*^c^*		NZB/B1NJ*^b^*
				NZO/HlltJ*^c^*
				NZW/LacJ*^c^*
				RF/J*^b^*
				ST/bJ*^b^*
				ZALENDE/EiJ*^c^*
				CAST/EiJ*^a^*

a Thomas *et al.* (2002).

b Jackson Laboratory website (https://www.jax.org/#).

c not previously reported.

The predicted AHR amino acid sequences were also compared with a multiple sequence alignment (Figure 5B). The imputation results coincide with previous reports that have shown that strain-specific polymorphisms lead to unique amino acid sequences. For example, as compared to the reference mouse genome, mice which carry an *Ahr^b2^*, *Ahr^b3^*, or an *Ahr^d^* allele have a variant (rs3021951) which negates a stop codon via an arginine substitution and, ultimately, leads to a longer open reading frame. SPRET/EiJ contain a 12 base insertion which leads to an addition of 4 amino acids. MOLF/EiJ *Ahr* sequence contain a 2 base insertion that leads to an additional 35 amino acids within the open reading frame. The imputation results also lead to a few novel findings as well. For example, PWK/PhJ grouped most closely with MOLF/EiJ, an *Ahr^b3^* strain, while aligning imputed genetic sequences. However, in aligning amino acid sequences, the PWK/PhJ aligned more closely with *Ahr*^b2^ and *Ahr*^d^ mice as compared to other *Ahr*^b3^ mice. While the PWK/PhJ carries some unique features of the *Ahr*^b3^ allele, such as a I808V substitution, this strain was not found to have insertions found in other *Ahr*^b3^ mice, such as MOLF/EiJ and SPRET/EiJ. Similarly, while polymorphisms are uniformly present within the genetic *Ahr* sequences across the mice which carry the *Ahr^b2^* and *Ahr^d^* alleles, the vast majority of mice carry identical amino acid sequences. For example, 9 of 15 and 16 of 17 mice which carry the *Ahr^b2^* and *Ahr^d^* allele, respectively, were predicted to have identical amino sequences. For both allelic groups, wild-derived strains (*i.e.*, CAST/EiJ, WSB/EiJ, and LEWES/EiJ) were found to carry the most amino acid sequence-altering polymorphisms.

## Discussion

Here, we report on MGP-Seq which was designed to impute polymorphism data from a gene of interest into the reference mouse genome. While other programs have been designed to impute gene sequences in the past (McLaren *et al.* 2016; Pabinger *et al.* 2014), this particular program has several key advantages including: a) requiring little computational experience and setup from user, b) requiring no data input from the user, and, finally, c) can be run across multiple strains simultaneously. The results can be used for a plethora of downstream analyses including QRTPCR primer design, multiple sequence alignments, protein modeling, and, overall, potentially aid in understanding how genetic alterations may alter activity of a gene, protein, or pathway of interest.

To outline the reproducibility and scalability of MGP-Seq, we report on imputation analysis of sequence variation in the murine aryl hydrocarbon receptor for 36 inbred mouse strains. For strains in which sequence data are available, MGP-Seq was found to correctly predict amino acid acid sequence and, thus, the allele carried. Notably, 11 of the 36 strains examined do not have *Ahr* sequences that have been previously characterized and, thus, are being predicted in this report for the first time. Comparing the alignments of the *Ahr* genetic and amino acid sequences across strain indicates the usefulness of MGP-Seq. In comparing the imputed gene and amino acid sequence that were predicted, the open-reading frames were found to encode highly conserved protein sequences. With that being said, the imputation results pick up several nonsynonymous substitutions within the murine *Ahr* that are of particular interest. For example, A375V substitution found in mice which carry the *Ahr*^d^ has been found to be critical for ligand-binding (Poland *et al.* 1994). Similarly, a SNP induces a premature stop codon that is unique the *Ahr^b1^* allele (Thomas *et al.* 2002). Notably, several other insertions are present which impact the amino acid sequence; for example, an insertion in the MOLF/EiJ results in an additional 35 amino acids within the open reading frame. However, several strains, such as the CAST/EiJ, WSB/EiJ, PWK/PhJ, were found to be unique in carrying several polymorphisms which are predicted to lead to different AHR amino acid sequences.

The MGP-Seq program has several limitations. First, the software is designed for imputation of inbred mouse strains and is currently designed to only query variants reported on the Mouse Genome Project website. While the software will not work with genomes that carry heterozygous alleles, such as Diversity Outbred stock mice, the software is scalable to impute sequences across larger numbers of inbred strain panels, such as the Collaborative Cross, as variant call data becomes more easily-accessible. Second, MGP-Seq was not designed to read-in raw genotyping data. Notably, other software, such as the widely-used Ensembl VEP (McLaren *et al.* 2016), are well-suited for *de novo* analysis of raw variant call data. Third, the genetic sequences imputed are dependent on SNP and polymorphism data. As all polymorphisms for each gene are not likely assessed or reported in each mouse strain, the imputed gene sequences will likely not be 100% identical to data derived from fully sequenced genomes. Fourth, the inferred amino acid sequences are based on alignment within the mRNA reported from the reference mouse genome. As such, the outputted amino acid sequence will be dependent on the RNA isoform chosen during imputation. Finally, it is important to note that sequences provided by MGP-Seq are predictions and, thus, need to be confirmed with sequencing data to ensure that they are accurate.

All in all, we believe that MGP-seq provides a tool for the scientific community to better predict how polymorphisms impact genetic and amino acid sequences of a gene of interest in a strain-specific manner. The software can also be used to screen a large number of mouse strains for variants in a gene of interest that may impact the results of future experiments. As such, the MGP-Seq tool can be used to a) choose strains that best fit the research at-hand and b) avoid strains which do not fit the experimental model required.
